# Feeding ecology and behavioral adaptations shape injury patterns in central European ants

**DOI:** 10.1093/beheco/arag055

**Published:** 2026-05-23

**Authors:** Melvin Kenneth Opolka, Alina Koeters, Erik Thomas Frank

**Affiliations:** Department of Animal Ecology and Tropical Biology, Biocenter, University of Würzburg, Am Hubland, 97074 Würzburg, Germany; Animal Population Ecology, Bayreuth Center for Ecology and Environmental Research (BayCEER), University of Bayreuth, Universitätsstraße 30, 95447 Bayreuth, Germany; Department of Animal Ecology and Tropical Biology, Biocenter, University of Würzburg, Am Hubland, 97074 Würzburg, Germany; Department of Animal Ecology and Tropical Biology, Biocenter, University of Würzburg, Am Hubland, 97074 Würzburg, Germany

**Keywords:** ant injuries, feeding ecology, foraging behavior, pitfall traps, myrmecology

## Abstract

Injuries are common in animals and represent a major threat to individual survival. They can result from inter- or intraspecific conflict, predation, or pugnacious prey. Despite their potential ecological and evolutionary importance, injury patterns remain poorly documented in animal populations. To test whether a species’ feeding ecology or habitat can predict injury patterns, we quantified injury rates and affected body regions among native ant species collected from different habitats in Bavaria, Germany. Specimens were sampled using pitfall traps, which proved to be an efficient method for injury assessment. Injury rates varied substantially among species and genera, ranging from 0% to 38%. Predatory ant species exhibited higher frequencies of leg injuries, whereas omnivorous species were more frequently injured at the antennae. The distribution of injuries likely reflects both foraging ecology and species-specific wound care behaviors, with a high frequency of trochanter injuries potentially indicating prior amputation events to cope with infected leg injuries, as observed in *Lasius alienus*. Our findings demonstrate that injury propensity and distribution are shaped by feeding habits and behavioral adaptations, providing comparative evidence that the costs and management of injuries vary systematically among ant species. Our study thus highlights injuries as a measurable axis of selection that may have contributed to the emergence of wound care and other forms of social immunity in ants.

## Teaser

Ants may be small, but their lives are full of danger. Some species regularly lose legs, others damage their antennae, all depending on how they make a living. By comparing European ants, we found that predators suffer more leg injuries, while omnivores more often injure their antennae. These injury patterns reveal how lifestyle shapes risks and suggest that coping with wounds might have been a key force in the evolution of ant wound care.

## Introduction

Injuries are widespread in animals and have severe consequences for their fitness. Causes of injuries can include predation and fights with conspecifics over territory, food, or mating partners (Huntingford and Turner 1987; Bowerman et al. 2010; de Ramirez et al. 2012). Injuries can lead to the loss of bodily fluids or impair an individual's movement while searching for food or mating partners (Beamish and O’Riain 2014; Krause et al. 2017). Ultimately, injuries also diminish both survival and reproductive success by increasing the risk of lethal infections (Carey et al. 2009; Moroń et al. 2012; Frank et al. 2023, 2024). Yet it is poorly understood how frequently injuries occur in nature and what adaptations animals have developed to mitigate their negative impacts.

Injury rates and related adaptations have mostly been studied at the level of individual species (Subasi et al. 2024; Pusceddu et al. 2025). Social animals are particularly noteworthy in this context, as they often exhibit behavioral responses to injuries. Primates are known to swallow unchewed leaves to expel intestinal parasites such as nematodes, as well as to alleviate inflammation and associated symptoms (Huffman and Seifu 1989; Wrangham 1995; Huffman 1997). In addition, plant-based or inorganic substances are frequently applied externally to the fur or on wounds for their antiparasitic, antibacterial, or anti-inflammatory effects (Baker 1996; Huffman 1997; Laumer et al. 2024).

The best-studied examples for injury care are in social insects, particularly in ants. Workers in the termite-hunting ant *Megaponera analis* rescue wounded nestmates during raids on termites by carrying them back to the nest (Frank et al. 2017). They can also identify infected wounds and treat them with antimicrobial compounds produced in their metapleural glands, thereby drastically reducing the mortality rate of treated nestmates (Frank et al. 2018, 2023). Wound care behaviors have also been observed in various other ant species. In generalist *Camponotus* ants workers amputate the limbs of injured individuals to stop pathogen spread (Frank et al. 2024; Fujimoto et al. 2025). Wound care has also been reported in the short-lived foragers of *Cataglyphis nodus*, which survive for only about 6 days as foragers (Beydizada et al. 2024). In *Formica cinerea*, injuries reduce survival, and injured workers showed increased rescue behavior toward intact nestmates (Turza and Miler 2023). Even in *Eciton burchellii*, where colonies can reach over 1 million ants, a 2-tiered wound care system has evolved, including first aid at the hunting site and antimicrobial wound care with the metapleural gland inside the bivouac (Lagos-Oviedo et al. 2025). This diversity in colony size, lifespan, natural history, and wound care behaviors across species highlights the remarkable adaptability and ubiquity of wound care strategies in ants. Theoretical models further underline their importance in increasing overall colony fitness under high injury rates (Lagos-Oviedo et al. 2025). Injury care therefore appears to be an overlooked factor affecting the ecological success of ants (Pull 2024).

Yet while the importance of injury management in ants has been well studied behaviorally, its ecological importance, the causes and likelihood of injuries remain poorly understood. In social insects, injuries most likely occur while foraging outside the nest, by predators, prey or competitors for food or nesting sites (Nonacs and Dill 1988; Lima 2002; Boulay et al. 2007; Mukherjee and Heithaus 2013; Frank et al. 2017). The life traits of ant species, including their habitat preferences and dietary habits, are therefore crucial determinants of their susceptibility to injuries. Omnivorous ant species could experience a lower risk of injury than predatory species because they rely more heavily on scavenging and trophobiosis, thus avoiding direct confrontations with pugnacious prey (McGlynn 2000). By contrast, predatory species actively subdue prey and may thus face a higher risk of physical damage during hunting, which could be reflected in elevated overall injury rates.

We further expected injury rates to vary across habitats, as ants living in more physically demanding or harsher environments may be more exposed to mechanical stress or greater competition over fewer food sources leading to more aggressive encounters (Gilad et al. 2022; Han et al. 2023). Behavioral differences may also influence which body parts are most often injured. Intra-species encounters often follow ritualized displays, involving close head-to-head contact, potentially leading to more antennal injuries. In contrast, predator-prey interactions are likely much more chaotic, resulting in potential severe injuries to all exposed extremities or even death of the predator (Lima Vieira et al. 2024).

The objective of this study is therefore to analyze injury rates among Central European ant species in Bavaria, Germany, to identify potential factors driving these injury rates, such as habitat preference, natural history or dietary habits. We hypothesize that predatory ant species will exhibit higher rates of injury compared with omnivorous ant species. Furthermore, we expect the distribution of injury types (leg or antenna) to differ between species depending on their natural history and the most likely injury sources.

## Materials and methods

### Study region and study sites

**Figure 1:**
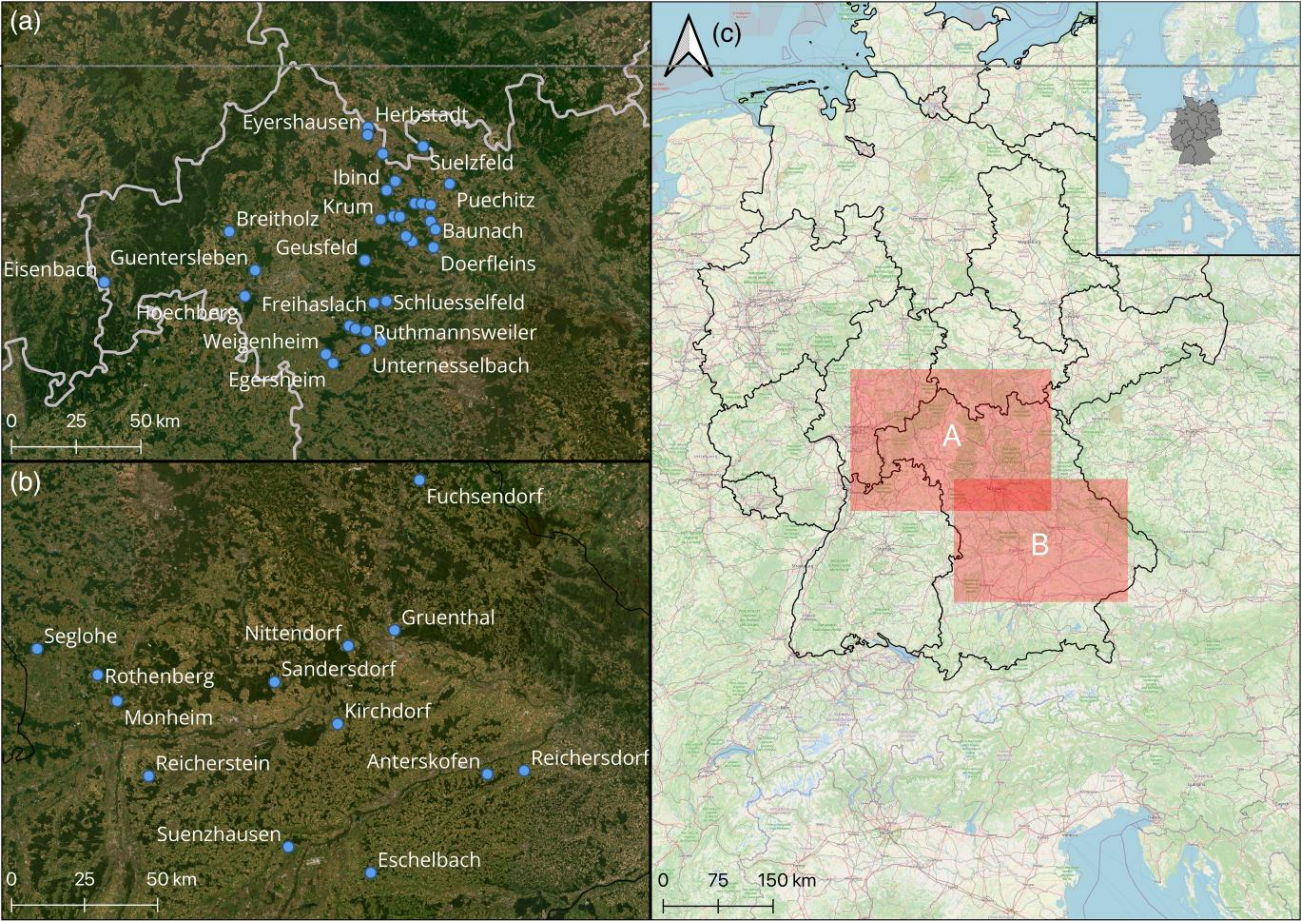
Study area in Bavaria, Germany with blue dots marking all plots used in this study. a) Thirty-one plots situated in the upper north of Bavaria (study region a). b) Thirteen plots are located further south in central Bavaria (study region b). c) Map of Germany with red areas highlighting the 2 study regions in Bavaria where the plots were located. The top right insert includes an overview of Germany (in gray) and its neighboring countries. Map created with QGIS 3.32.3-Lima using ESRI “Satellite” basemap (ESRI, 2019).

The study was conducted in May 2022 at 44 sites throughout Bavaria (Germany) covering a region of approximately 45,000 km^2^ (Fig. 1). Most plots were on Keuper formation, a fertile soil type with excellent water retention properties (Böse et al. 2018). The average temperature in the region for the period from 2003 to 2023 varied between 8.4 °C and 11.2 °C and precipitation ranged between 413 and 792 mm for the same period (Kaspar et al. 2013). The sites used in this study can be divided into 4 habitat types: grassland, forest edge, slope and wooded strip. In total, we had 10 plots with grasslands, 17 plots with forest edges, 9 plots with slopes and 8 plots with wood strips. Grasslands were defined as open landscapes characterized by poor soils and low-growing vegetation (Figure S1e). The forest edge was the transitional area between a forest and adjacent open habitats such as meadows or fields (Figure S1c and g). A slope was defined as the terrain along an incline where the land either rises or falls (Figure S1a and f). The habitat wood strip was a narrow section of wooded land that often runs along fields or pathways (Figure S1b and d).

### Ant community sampling

For each plot, 5 sampling points were created in a line with 1 m between each other. On each sampling point, a pitfall trap was placed, buried at soil level, and filled with a 1:2 propylene glycol-water solution, with a perfume-free detergent. The pitfall traps had a diameter of 82 mm and were recollected after 2 weeks. This resulted in 220 sampling points which were pooled on plot level after sampling (5 traps per plot, across 44 plots).

### Ant identification and quantification of the injury rate

Pitfall trap samples were transported to the Department of Animal Ecology and Tropical Biology (Zoology III) at Julius-Maximilians University of Würzburg. All ants were transferred to 70% ethanol for later identification. Identification of the ants was done to species-level, based on keys provided by Lebas et al. (2019) and Seifert (2007) (Table S1). Each species was then assigned to 1 of 3 types of feeding ecology: omnivore, predator, and trophobiont (Table S2). All samples were stored at the University of Würzburg (Germany). The injury rate was quantified by using a binocular (Nikon, SMZ645) with 6.7–50× magnification, to verify the state of all extremities of every ant. An injury was defined as either a complete or partially severed extremity (Figure S2). During sample processing, we took great care to avoid breaking extremities. Only genuine injuries were counted; any detached extremities found in the samples were identified as processing artifacts and excluded from the injury counts. For each observed injury, we recorded the location (front, middle, hind leg or antenna), side (left or right), and the specific injury site (funiculus, scapus, trochanter, femur, tibia or tarsus). Injury rate was then calculated as the number of injuries divided by the total number of ants per species and pitfall.

### Hand-collected injury rate

To verify that our sampling method with pitfalls is valid for identifying injuries in ants, we compared our injury rate of ants sampled with pitfalls with that of ants that have been collected by hand. In June 2024, approximately 100 foraging workers from 10 different colonies of *Lasius niger* were hand-collected and stored in 70% ethanol. After the hand collection, identical pitfall traps, constructed as before, were placed next to the same 10 *L. niger* colonies for 2 weeks, and the injury rate of the trapped workers was quantified as described above.

### Statistical analysis

For statistical analyses and graphical illustration, we utilized the R software (version 4.4.2) in combination with the RStudio interface (version 2024.12.0 + 467) and the *ggplot2* package (version 3.4.4) (Wickham and Chang 2016). Our dataset contained many species that were only found a few times. To reduce the influence of very small samples, only records with at least 5 individuals were retained. To increase the robustness of our injury rate estimates, we excluded all ant species for which fewer than 200 individuals were collected, and which occurred in <6 plots. These criteria reduced the number of species analyzed from 34 to 8. Data were first tested for normality using the Shapiro-Wilk test and visualized with Q-Q plots. Normality was not met for the variable's *habitat, ant species, genus, body side,* and *feeding ecology*, so Kruskal–Wallis tests were performed to assess differences among groups. To check for differences between injuries occurring on the left and right sides of ants, a Wilcoxon rank sum test was conducted. To analyze factors influencing injury rates, we fitted linear mixed-effects models (LMMs) using the *lme4* package (version 1.1) (Bates et al. 2015). The primary model assessed the effects of feeding ecology and habitat on injury rate, including location as a random effect to account for spatial variation.

To evaluate whether the observed effect of feeding ecology was robust to species-level structure, we fitted an additional mixed-effects model including ant species identity as a second random intercept. For the phylogenetic analysis, species names were matched to Open Tree Taxonomy identifiers using the *rotl* package (Michonneau et al. 2016), and an induced subtree for the focal taxa was obtained. Tip labels were standardized to match the dataset, and branch lengths were assigned using Grafen's method. The resulting tree was used in a phylogenetic mixed model fitted with the package *phyr* (Li et al. 2020). Model selection was based on Akaike's Information Criterion (AIC). Furthermore, we applied an LMM assessing the influence of body part and feeding ecology on injury rates, with the location as a random effect. Model assumptions were verified by checking residual distributions using the package *DHARMa* version 0.4.7 (Hartig 2018). Post-hoc comparisons were conducted using Dunn's test with Bonferroni correction for multiple comparisons using the FSA package version 0.6.9 (Ogle et al. 2017). We conducted Wilcoxon rank sum tests to examine how the sampling method affected injury rates.

## Results

**Figure 2:**
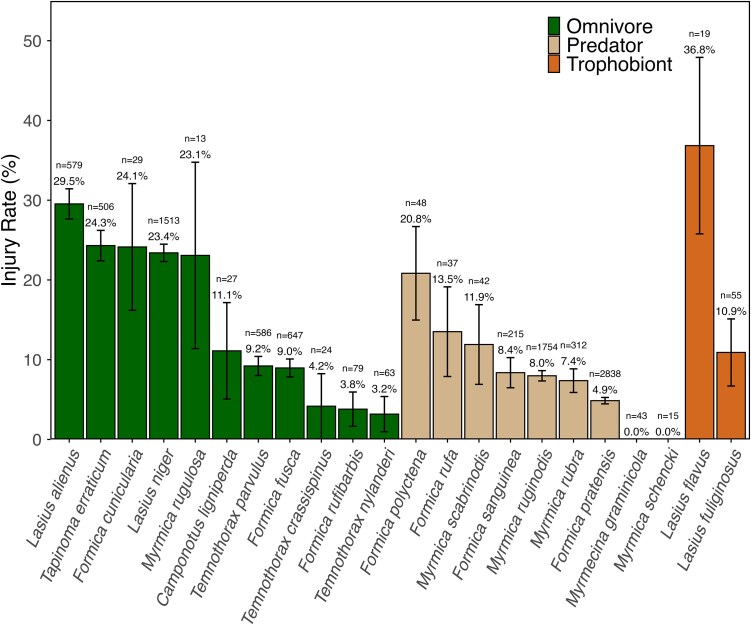
All ants found in the 44 plots, along with their injury rates and sample sizes for each species, given above the bars. Error lines indicate the binomial standard error of injury rates.

The 220 pitfall traps, distributed across the 44 plots, collected 9,475 ants from 3 subfamilies, belonging to 12 genera, for a total of 34 ant species (Fig. 2). The mean number of ants caught in each plot was 214.6 (± 248.9 SD, *n* = 44). Overall, 1,137 of the 9,475 ants (12%) were injured at least once. The most common ant genera in our study were *Formica*, *Lasius* and *Myrmica*, with *Myrmica ruginodis* being by far the most common ant species, occurring at 42 of 44 sampling sites (Table S1). While the slope habitat had a slightly higher Shannon diversity index (H′) than the other habitats (slope: H′ = 2.16; woodstrip: H′ = 2.11; grassland: H′ = 2.05; forest edge: H′ = 2.00), these differences were not significant (χ^2^ = 3, df = 3, *P* = 0.39).

After sorting out the rare species, we counted 983 ants with injuries, for a global injury rate of 15.8% (*n* = 8,205 individuals) for the remaining 8 most common ant species studied. A total of 747 ants (76%) had only 1 injury, 179 (18%) had 2 injuries, and 58 (6%) were injured more than twice. The average injury rate per plot was 16.5% for these 8 species (± 8.7 SD, *n* = 44).

**Figure 3:**
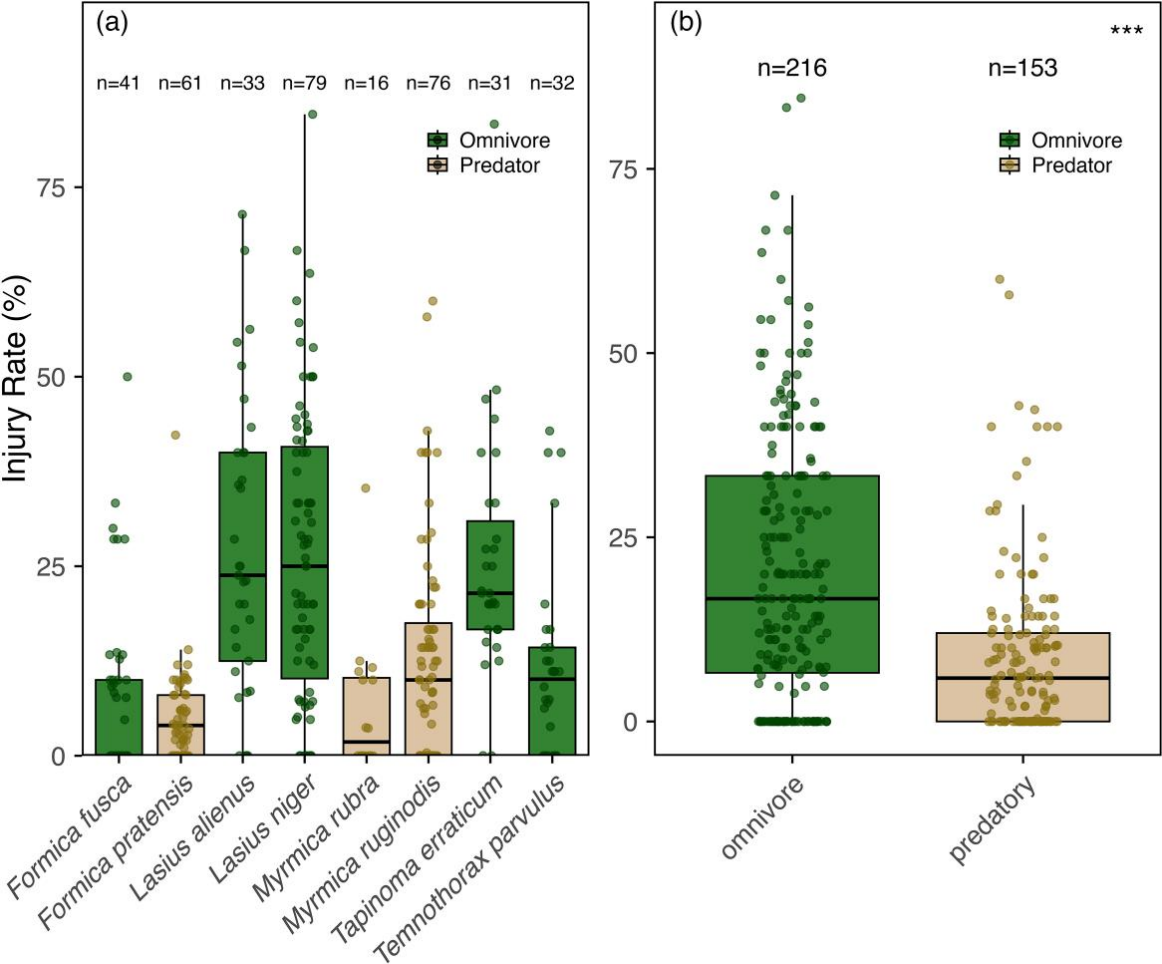
Filtered dataset showing the eight species with at least 200 sampled individuals and occurrences in more than five plots. Sample sizes are shown above the boxes. a) Injury rates for the eight most common ant species, with each point representing one pitfall trap. b) Pooled injury rates for omnivorous and predatory species shown in A. Omnivorous species exhibit significantly higher injury rates than predatory species (LMM, Estimate = -10.84 ± 3.87, t = -2.81, *p* = 0.006). Each point represents the injury rate per species and pitfall.

The injury rate varied significantly across species. A high injury rate was observed in *Lasius alienus* (23.1%), *Lasius niger* (25%) and *Tapinoma erraticum* (21.4%). In contrast, the wood ant *Formica pratensis* (4%) and *Temnothorax parvulus* (10.1%) showed a low injury rate (Fig. 3a).

**Figure 4:**
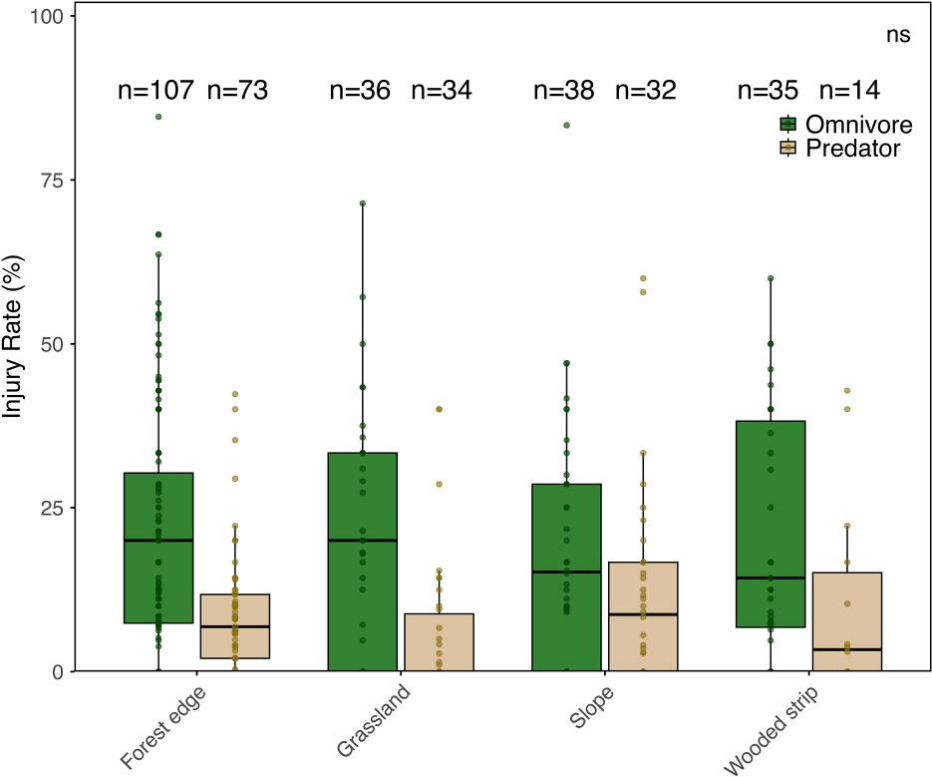
Filtered dataset showing injury rates in our four sampled habitat types. Each data point represents the injury rate in a single pitfall for each species. Sample size is indicated above the boxes.

Injuries were equally likely to occur on the left or right side of the body (Wilcoxon rank sum test: W = 68,693; *P* = 0.83). Model comparison based on AIC for the causes of injury rates (eg feeding ecology and habitat) indicated that the reduced model, which included only feeding ecology as a fixed effect, provided the best fit, while habitat and its interaction with feeding ecology showed no significant influence on injury rates (all *P* > 0.1; Fig. 4, Table S3). Predatory species exhibited lower injury rates (9 ± 11.6%, *n* = 4,698 ants) compared with omnivorous species (20.7 ± 18.4%, *n* = 3,507 ants; LMM: Estimate = −11.20 ± 1.80, *t* = −6.22, *P* < 0.05; Fig. 3b). In the phylogenetic mixed model including the species tree, feeding ecology was not significantly associated with injury rate (Estimate = −5.96 ± 5.07, *z* = −1.17, *P* = 0.24; Table S5), suggesting that part of the observed pattern may reflect phylogenetic structure.

**Figure 5:**
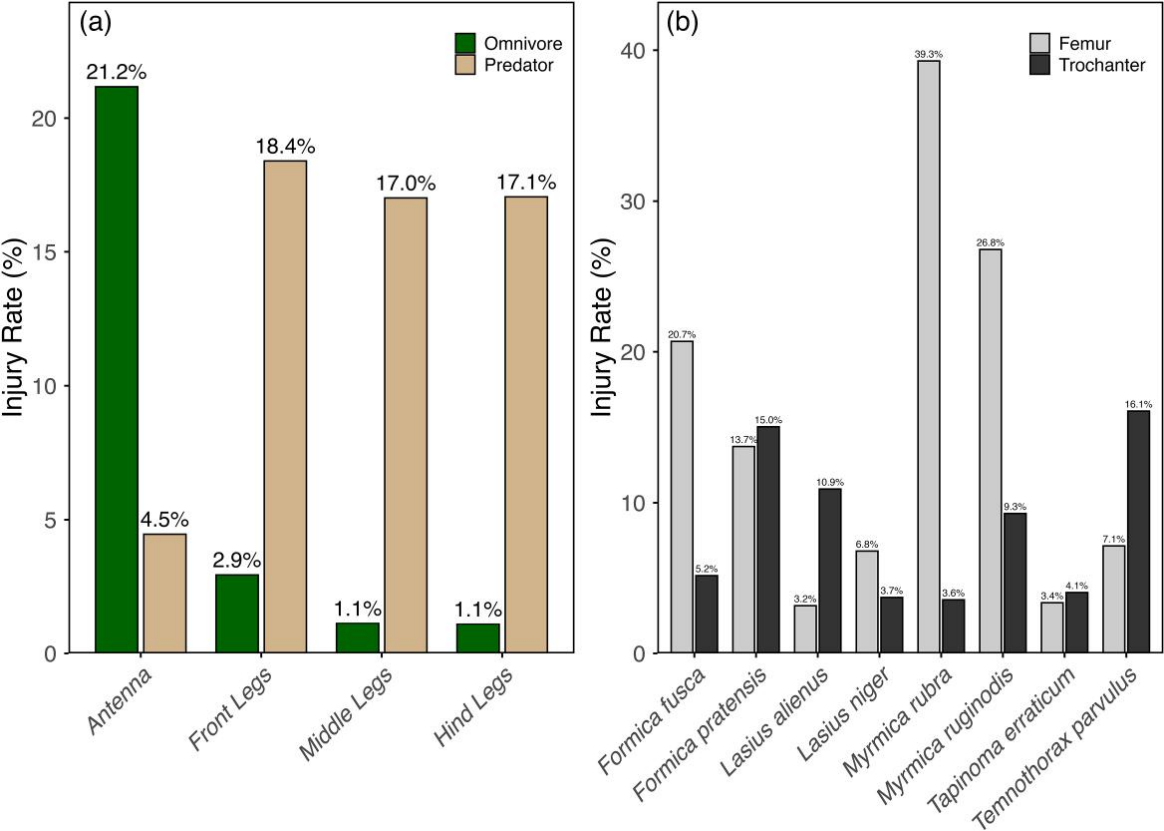
Filtered dataset showing mean injury rates (%) at a) different body part regions of omnivorous and predatory ant species. The total number of injuries studied was *n* = 1202 in 983 injured ants, as some ants had multiple injuries. The bars are divided into feeding ecologies of ant species, with omnivorous species (*n* = 702) and predatory species (*n* = 281). b) Average Injury rates on the trochanter and femur for the 8 most abundant ant species in our study (*n* = 240 injuries).

Injury rates differed significantly across body parts and between feeding ecology strategies, with a significant interaction between both factors (LMM: feeding ecology × body part, all *P* < 0.001; Table S4). Omnivorous ants exhibited higher rates of antennal injuries, while predatory ants showed relatively more injuries on the legs. Post-hoc comparisons confirmed significantly higher injury rates on the antennae than at any leg pair when analyzing the pooled dataset of omnivorous and predatory species (Antenna vs. Front Legs: *t* = 13.77, *P* < 0.0001; Antenna vs. Middle Legs: *t* = 15.17, *P* < 0.0001; Antenna vs. Hind Legs: *t* = 14.52, *P* < 0.0001; Tukey-adjusted pairwise comparisons; Fig. 5a), while no significant differences were found in the injury rates across the leg pairs themselves (all *P* > 0.05). Nevertheless, the injury distribution varied significantly among ant species (Chi^2^, χ^2^ = 229.69, df = 21, *P* < 0.0001), indicating that some species experienced disproportionate injury rates in specific body regions. Notably, this effect was particularly evident when examining injuries on the trochanter and femur. Some species displayed a relatively equal distribution of injuries between the 2 segments, whereas others exhibited much higher injury rates concentrated on either the trochanter or the femur (Fig. 5b).

Ultimately, to test whether injuries occurred because of the pitfall traps, we compared injury rates among *Lasius niger* workers collected from 10 pitfall traps with those collected by hand at the same location. There was no significant difference between the 302 workers captured with the pitfall trap (injury rate 34 ± 16.1%, *n* = 10) compared with the hand-collected 1,004 workers (21.8 ± 7.9%, *n* = 10; *t*-test: *t* = −2.15, df = 13.1, *P* = 0.051; Figure S3a). Even when considering the data on the 1,513 *Lasius niger* workers from the original data set (injury rate 26.3% ± 19.1%, *n* = 79), there was no significant difference between the methods (Kruskal–Wallis test: χ^2^ = 2.99, df = 2, *P* = 0.224; Figure S3). Across sampling methods, antennal injuries consistently comprised a high proportion of total injuries: 80.6% (87 out of 108 injuries) in pitfall re-sampling, 88.1% (193 out of 219 injuries) in hand-collected ants, and 87.5% (293 out of 335 injuries) in pitfall main study samples. A Kruskal–Wallis test with post-hoc Dunn test revealed a significant difference only in antennal injury rates between pitfall individuals from the main study and resampled individuals (χ^2^ = 7.83, df = 2, *P* = 0.04; Figure S3b).

## Discussion

This is the first study to quantify injury rates of ant populations across different habitats. We show how simple pitfall traps can be used to quantify these injury rates, a cost-effective and time-efficient approach. Injuries occurred across all species but followed distinct patterns related to species feeding ecologies: predatory species had a higher rate of leg injuries, while omnivorous ants showed significantly more antenna injuries.

Differences in injury rates and locations in omnivorous ants could be explained by ritualized confrontations that typically involve head-to-head interactions (van Wilgenburg et al. 2005; Lima Vieira et al. 2024). During these close-range displays, the antennae are directly exposed and therefore particularly prone to injury. Omnivorous ant species, which do not actively hunt prey, may thus have a lower risk of leg injuries due to the lack of high-risk interactions with prey. However, their dependence on resources such as honeydew brings them into frequent contact with both conspecifics and other ant species (McGlynn 2000). As a result, more frequent ritualized or direct confrontations may help explain the elevated antennal injury rates observed in these species. However, such behaviors are not exclusive to nonpredatory ants and are also reported in predatory lineages such as army ants (Yao 2014; Baudier and Pavlic 2020).

While differences in injury location can be explained by the type of interactions typical for omnivorous species, their overall higher injury rates were unexpected. Contrary to our hypothesis, omnivorous ants showed higher injury frequencies than predatory species, which were expected to be more frequently or severely injured due to their aggressive encounters with prey (Frank et al. 2017; Lagos-Oviedo et al. 2025). A possible explanation is that omnivorous species in our study are generally smaller and less well armed (Seifert 2007), making them more vulnerable during interspecific conflicts. While predatory species usually have a thicker cuticle that makes injuries less likely compared with the thinner cuticle of omnivorous Formicinae (Peeters et al. 2017). As a result, predatory species may exhibit fewer visible injuries overall, while in Formicinae, even a single aggressive encounter could carry a higher risk of losing an extremity. This interpretation is also consistent with our analyses, in which the effect of feeding ecology weakened after accounting for ant species and disappeared in the phylogenetic model, suggesting that the observed differences may be partly driven by species-specific behaviors and evolutionary traits rather than feeding ecology alone.

Injury rates were the same across all 4 habitat types, a pattern that was also consistent across both feeding ecology groups. Further underscoring the importance of species physiology and behavior in shaping injury frequencies. This pattern is in line with recent work proposing that injury risk and lethality largely arise from biotic interactions such as predator-prey interactions or competition, rather than colony dynamics or structural habitat factors (Frank et al. 2017; Frank and Linsenmair 2017; Rennolds and Bely 2023; Lagos-Oviedo et al. 2025).

Methodological factors should also be considered when interpreting injury patterns, as pitfall traps may introduce biases if injured ants are more likely to fall into the traps or if additional injuries arise from agonistic interactions within traps. When comparing hand-collected ants to pitfall trap samples we found no differences in injury composition. But ants from pitfall traps in the main study exhibited slightly higher injury rates to their antennae than those from the re-sampling, which may indicate a minor methodological effect or the selective capture of previously injured individuals. However, the overall injury rates remained consistent across all sampling methods, and in every case, injuries to the antennae clearly outnumbered those to the legs. This suggests that any method-related increase in injuries was likely minor and is unlikely to have influenced the main conclusions of our study. This finding, however, is based solely on *L. niger*, and it remains uncertain whether similar patterns would hold for other species.

Importantly, injury rates do not necessarily reflect injury risks in a population but rather the post-injury survival of injured workers. This survivorship bias is also not necessarily evenly distributed across species. Species with highly efficient rescue and wound care behaviors could exhibit a much higher injury rate, even with relatively low injury risks. Conversely, species that have a higher likelihood of lethal injuries, could exhibit a much lower injury rate, even though they have a high injury risk.

This survivorship bias in injury rates and its distribution may thus also offer indications on the presence of wound care behaviors. A high injury rate among foragers not only suggests frequent exposure to injury risks but also implies that at least some individuals successfully recover from injuries, potentially through wound care, since injuries observed in the field are likely from previous foraging trips. Recent theoretical models propose that high injury rates favor the evolution of wound care behaviors, independent of colony size, making injury rates the main predictor for the presence of wound care behaviors (Frank et al. 2017, 2018; Lagos-Oviedo et al. 2025). Our study adds to this by showing how such patterns may be shaped by ecological factors such as habitat or diet.

Moreover, the distribution of injuries can also indicate how injuries are managed in a species. Amputations at the trochanter by nestmates on ants with femur injuries have been reported in *Camponotus* species as an effective response to reduce infection risk (Frank et al. 2024; Fujimoto et al. 2025). If legs are amputated in a species, we would expect to see a decrease in the proportion of femur injuries and an increase in trochanter injuries. Our results indicate that *Lasius alienus* and *Temnothorax parvulus* showed a 3-fold higher rate of injuries at the trochanter than at the femur, whereas in most other species the opposite trend was observed. Interestingly, historical observations from the 1950s (Nachtwey 1950) documented 1 amputation event in *L. alienus*, supporting the interpretation that *L. alienus* could use amputations as a behavioral response to injury and infection-related challenges.

## Conclusion

This study highlights that differences in injury rates and affected body regions among Central European ant species are shaped by feeding ecology characteristics such as diet and behavior. Predatory species were more prone to leg injuries, while omnivorous species showed higher rates of antennal injuries, reflecting differences in foraging risks. Furthermore, the distribution of injuries could provide information on a species’ wound response strategies, such as leg amputations. Importantly, our findings demonstrate that pitfall traps are an effective tool for assessing injury rates across multiple species under natural conditions. Ultimately, our study highlights the ubiquity of injuries in ants, making it an important and measurable axis of selection that may have contributed to the emergence of wound care and other forms of social immunity.

## Supplementary Material

arag055_Supplementary_Data

## Data Availability

Analyses reported in this article can be reproduced using the data and R script provided by Opolka et al. (2026).

## References

[arag055-B1] Baker M . 1996. Fur rubbing: use of medicinal plants by capuchin monkeys (*Cebus capucinus*). Am J Primatol. 38:263–270. 10.1002/(SICI)1098-2345(1996)38:3<263::AID-AJP5>;3.0.CO;2-X.

[arag055-B2] Bates D, Mächler M, Bolker B, Walker S. 2015. Fitting linear mixed-effects models using lme4. J Stat Softw. 67:1–48. 10.18637/jss.v067.i01.

[arag055-B3] Baudier KM, Pavlic TP. 2020. Incidental interactions among Neotropical army-ant colonies are met with self-organized walls of ants (Hymenoptera: Formicidae). Myrmecol News. 30:251–258. 10.25849/MYRMECOL.NEWS_030:251.

[arag055-B4] Beamish EK, O’Riain MJ. 2014. The effects of permanent injury on the behavior and diet of commensal chacma baboons (*Papio ursinus*) in the Cape Peninsula, South Africa. Folia Primatol (Basel). 35:1004–1020. 10.1007/s10764-014-9779-z.

[arag055-B5] Beydizada N, Abels A, Schultheiss P, Frank E. 2024. Injury-dependent wound care behavior in the desert ant *Cataglyphis nodus*. Behav Ecol Sociobiol. 78, article number 97. 10.1007/s00265-024-03511-1.

[arag055-B6] Böse M, Ehlers J, Lehmkuhl F. 2018. Land und Meer im Wandel—Norddeutschland bevor die Eiszeit kam. In: Böse M, Ehlers J, Lehmkuhl F, editors. Deutschlands Norden: Vom Erdaltertum zur Gegenwart. Springer. p. 21–39. 10.1007/978-3-662-55373-2_2.

[arag055-B7] Boulay R, Cerdá X, Simon T, Roldan M, Hefetz A. 2007. Intraspecific competition in the ant *Camponotus cruentatus*: should we expect the ‘dear enemy’ effect? Anim Behav. 74:985–993. 10.1016/j.anbehav.2007.02.013.

[arag055-B8] Bowerman J, Johnson PTJ, Bowerman T. 2010. Sublethal predators and their injured prey: linking aquatic predators and severe limb abnormalities in amphibians. Ecology. 91:242–251. 10.1890/08-1687.1. 20380213

[arag055-B9] Carey JR et al 2009. Leg impairments elicit graded and sex-specific demographic responses in the tephritid fruit fly *Anastrepha ludens*. Exp Gerontol. 44:541–545. 10.1016/j.exger.2009.05.006. 19457447 PMC2730757

[arag055-B10] de Ramirez SS, Hyder AA, Herbert HK, Stevens K. 2012. Unintentional injuries: magnitude, prevention, and control. Annu Rev Public Health. 33:175–191. 10.1146/annurev-publhealth-031811-124558. 22224893

[arag055-B11] Frank ET et al 2017. Saving the injured: rescue behavior in the termite-hunting ant *Megaponera analis*. Sci Adv. 3:e1602187. 10.1126/sciadv.1602187. 28439543 PMC5389746

[arag055-B12] Frank ET et al 2023. Targeted treatment of injured nestmates with antimicrobial compounds in an ant society. Nat Commun. 14:8446. 10.1038/s41467-023-43885-w. 38158416 PMC10756881

[arag055-B13] Frank ET et al 2024. Wound-dependent leg amputations to combat infections in an ant society. Curr Biol. 34:3273–3278.e3. 10.1016/j.cub.2024.06.021. 38959879

[arag055-B14] Frank ET, Linsenmair KE. 2017. Saving the injured: evolution and mechanisms. Commun Integr Biol. 10:e1356516. 10.1080/19420889.2017.1356516. 29260800 PMC5731505

[arag055-B15] Frank ET, Wehrhahn M, Linsenmair KE. 2018. Wound treatment and selective help in a termite-hunting ant. Proc R Soc Lond B Biol Sci. 285:20172457. 10.1098/rspb.2017.2457. PMC5829198 29445019

[arag055-B16] Fujimoto S et al 2025 Better Safe Than Sorry: Leg Amputations as a Prophylactic Wound Care Behaviour in Carpenter Ants. Proc Biol Sci. 292: 20251688. 10.1098/rspb.2025.1688. PMC12539950 41119996

[arag055-B17] Gilad T, Dorfman A, Subach A, Scharf I. 2022. Leg or antenna injury in *Cataglyphis* ants impairs survival but does not hinder searching for food. Curr Zool. 68:441–450. 10.1093/cz/zoab027. 36090143 PMC9450180

[arag055-B18] Han S, Phillips BL, Elgar MA. 2023. Colony-level aggression escalates with the value of food resources. BMC Ecol Evol. 23:18. 10.1186/s12862-023-02117-x. 37193951 PMC10189932

[arag055-B19] Hartig F. 2018. *DHARMa: Residual Diagnostics for Hierarchical (Multi-Level/Mixed) Regression Models*, R package version 0.4.7. https://CRAN.R-project.org/package=DHARMa.

[arag055-B20] Huffman MA . 1997. Current evidence for self-medication in primates: a multidisciplinary perspective. Am J Phys Anthropol. 104(Suppl. 25):171–200. 10.1002/(SICI)1096-8644(1997)25+<171::AID-AJPA7>3.0.CO;2-7.

[arag055-B21] Huffman MA, Seifu M. 1989. Observations on the illness and consumption of a possibly medicinal plant *Vernonia amygdalina* (Del.), by a wild chimpanzee in the Mahale Mountains National Park, Tanzania. Primates. 30:51–63. 10.1007/BF02381210.

[arag055-B22] Huntingford FA, Turner AK. 1987. Conflict in the animal world. In: Huntingford FA, Turner AK, editors. Animal conflict. Springer Netherlands. p. 3–12. 10.1007/978-94-009-3145-9_1.

[arag055-B23] Kaspar F et al 2013. Monitoring of climate change in Germany—data, products and services of Germany's National Climate Data Centre. Adv Sci Res. 10:99–106. 10.5194/asr-10-99-2013.

[arag055-B24] Krause J et al 2017. Injury-mediated decrease in locomotor performance increases predation risk in schooling fish. Philos Trans R Soc Lond B Biol Sci. 372:20160232. 10.1098/rstb.2016.0232. 28673910 PMC5498294

[arag055-B25] Lagos-Oviedo JJ, Rajendra D, Gokhale CS, Schmitt T, Frank ET. 2025. Evolution of first aid and social wound care in an army ant society [preprint]. bioRxiv 677721. 10.1101/2025.09.22.677721.

[arag055-B26] Laumer IB et al 2024. Active self-treatment of a facial wound with a biologically active plant by a male Sumatran orangutan. Sci Rep. 14:8932. 10.1038/s41598-024-58988-7. 38698007 PMC11066025

[arag055-B27] Lebas C, Galkowski C, Blatrix R, Wegnez P. 2019. Die Ameisen Europas. Haupt Verlag.

[arag055-B28] Li D, Dinnage R, Nell LA, Helmus MR, Ives AR. 2020. Phyr: an r package for phylogenetic species-distribution modelling in ecological communities. Methods Ecol Evol. 11:1455–1463. 10.1111/2041-210X.13471.

[arag055-B29] Lima SL . 2002. Putting predators back into behavioral predator–prey interactions. Trends Ecol Evol. 17:70–75. 10.1016/S0169-5347(01)02393-X.

[arag055-B30] Lima Vieira ME, Teseo S, Azevedo DLO, Châline N, Araújo A. 2024. Competition through ritualized aggressive interactions between sympatric colonies in solitary foraging neotropical ants. Naturwissenschaften. 111:4. 10.1007/s00114-024-01891-y. 38289402

[arag055-B31] McGlynn TP . 2000. Do Lanchester's laws of combat describe competition in ants? Behav Ecol. 11:686–690. 10.1093/beheco/11.6.686.

[arag055-B32] Michonneau F, Brown JW, Winter DJ. 2016. Rotl: an R package to interact with the Open Tree of Life data. Methods Ecol Evol. 7:1476–1481. 10.1111/2041-210X.12593.

[arag055-B33] Moroń D, Lenda M, Skórka P, Woyciechowski M. 2012. Short-lived ants take greater risks during food collection. Am Nat. 180:744–750. 10.1086/668009. 23149399

[arag055-B34] Mukherjee S, Heithaus MR. 2013. Dangerous prey and daring predators: a review. Biol Rev Camb Philos Soc. 88:550–563. 10.1111/brv.12014. 23331494

[arag055-B35] Nachtwey R . 1950. Instinkt, Rätsel der Welt: Lebensbilder aus Wald und Flur. Brockhaus.

[arag055-B36] Nonacs P, Dill LM. 1988. Foraging response of the ant *Lasius pallitarsis* to food sources with associated mortality risk. Insectes Soc. 35:293–303. 10.1007/BF02224061.

[arag055-B37] Ogle D, Ogle MD. 2017. Package ‘FSA’. CRAN Repos:1–206. https://github.com/fishR-Core-Team/FSA.

[arag055-B38] Opolka MK, Koeters A, Frank E. 2026. Feeding Ecology and Behavioral Adaptations shape Injury Patterns in Central European Ants [Data set]. Zenodo. 10.5281/zenodo.19910014. PMC13247590 42273019

[arag055-B39] Peeters C, Molet M, Lin C-C, Billen J. 2017. Evolution of cheaper workers in ants: a comparative study of exoskeleton thickness. Biol J Linn Soc Lond. 121:556–563. 10.1093/biolinnean/blx011.

[arag055-B40] Pull CD . 2024. Social evolution: limb amputation prevents infection in ants. Curr Biol. 34:R677–R679. 10.1016/j.cub.2024.05.047. 39043138

[arag055-B41] Pusceddu M et al 2025. Animal medical systems from Apis to apes: history, recent advances and future perspectives. Biol Rev Camb Philos Soc. 100:2608–2624. 10.1111/brv.70060. 40717357 PMC12586310

[arag055-B42] Rennolds CW, Bely AE. 2023. Integrative biology of injury in animals. Biol Rev Camb Philos Soc. 98:34–62. 10.1111/brv.12894. 36176189 PMC10087827

[arag055-B43] Seifert B . 2007. Die Ameisen Mittel- und Nordeuropas. Lutra.

[arag055-B44] Subasi BS, Grabe V, Kaltenpoth M, Rolff J, Armitage SAO. 2024. How frequently are insects wounded in the wild? A case study using *Drosophila melanogaster*. R Soc Open Sci. 11:240256. 10.1098/rsos.240256. 39100166 PMC11296199

[arag055-B45] Turza F, Miler K. 2023. Injury shortens life expectancy in ants and affects some risk-related decisions of workers. Anim Cogn. 26:1643–1647. 10.1007/s10071-023-01810-0. 37450227 PMC10442280

[arag055-B46] van Wilgenburg E, van Lieshout E, Elgar MA. 2005. Conflict resolution strategies in meat ants (Iridomyrmex purpureus): Ritualised displays versus lethal fighting. Behaviour. 142:701–716. 10.1163/1568539054729150.

[arag055-B47] Wickham H, Chang W. 2016. *ggplot2: An implementation of the Grammar of Graphics*.

[arag055-B48] Wrangham RW . 1995. Relationship of chimpanzee leaf-swallowing to a tapeworm infection. Am J Primatol. 37:297–303. 10.1002/ajp.1350370404. 31936954

[arag055-B49] Yao I . 2014. Costs and constraints in aphid-ant mutualism. Ecol Res. 29:383–391. 10.1007/s11284-014-1151-4.

